# A comparison of ABR and ASSR using narrow-band-chirp-stimuli in children with single-sided deafness of various etiology

**DOI:** 10.1007/s00405-025-09708-y

**Published:** 2025-10-01

**Authors:** Donata Gellrich, Daniel Polterauer, Sophia Stoecklein, Patrick Huber, Tobias Rader, Katharina Eder

**Affiliations:** 1 Department of Otorhinolaryngology, Head and Neck Surgery, Division of Phoniatrics and Pediatric Audiology, University Hospital, Ludwig-Maximilians-University Munich (LMU), Marchioninistr. 15, 81377 Munich, Germany; 2 Department of Otorhinolaryngology, Head and Neck Surgery, Division of Audiology, University Hospital, Ludwig-Maximilians-University Munich (LMU), Munich, Germany; 3 Department of Radiology, University Hospital, Ludwig-Maximilians-University Munich (LMU), Munich, Germany; 4 Department of Pediatric Audiology, kbo-Kinderzentrum München gemeinnützige GmbH, Heiglhofstr. 69, 81377 München, Germany

**Keywords:** ASSR, ABR, Single-sided deafness, Children, Malformation, Cochlear nerve

## Abstract

**Purpose:**

Auditory steady-state responses (ASSR) are available for frequency-dependent hearing threshold estimation in addition to the technique of conventional auditory brainstem responses (ABR). Although ABR and ASSR principally show strong correlations in hearing threshold estimation, there is preliminary evidence that temporal bone malformations might be associated with significantly greater differences between ABR- and ASSR-results. Therefore, the present study aimed to compare hearing threshold estimation derived from ABR and ASSR in a larger cohort of single-sided deafness (SSD) of various etiology, including temporal bone anomalies.

**Methods:**

The diagnostic consistency between ABR and ASSR using narrow-band-chirp-stimuli at 1000, 2000, and 4000 Hz was analyzed in 47 children with single-sided deafness with varying MRI-morphologic findings: cochlear nerve malformation (CNM, *n* = 24), cochlear malformation (CM, *n* = 7) vs. combined malformation (CM + CNM, *n* = 8) vs. absent temporal bone and inner ear pathology (*n* = 8). Children with additional health issues other than SSD were excluded.

**Results:**

ABR and ASSR showed a strong correlation in deaf ears without malformation (*r* = 0.728, *p* < 0.0001), a moderate correlation in isolated cochlear malformation (*r* = 0.574, *p* = 0.01), and a weak correlation in case of cochlear nerve anomaly (*r* = 0.189, *p* = 0.112 in CNM and *r* = 0.235, *p* = 0.268 in CM + CNM). Ears with isolated CNM showed an average discrepancy of 23.40 ± 15.19 dB, *p* < 0.00001 between ABR and ASSR (vs. 17.08 ± 15.81 dB, *p* = 0.0008 in CNM + CM, vs. 7.63 ± 8.56 dB, *p* = 0.008 in CM, vs. 4.38 ± 4.96 dB, *p* = 0.036 in ears without malformation and vs. 0.36 ± 4.75 dB, *p* = 0.748 in healthy control ears). In ears with highly discrepant ASSR and ABR values, enlarged ABR wave I and otoacoustic emissions were frequently present.

**Conclusion:**

In cochlear nerve malformation, ASSR and ABR frequently provide significantly discrepant hearing threshold estimations, probably derived from a cochlear origin. ASSR should only be used in conjunction with conventional ABR in the diagnostic management of suspected severe-profound hearing loss or deafness in children. A large difference between ASSR and ABR thresholds may indicate a cochlear nerve anomaly.

## Introduction

Frequency-specific auditory brainstem responses (ABR) have become an indispensable diagnostic tool for objective hearing threshold estimation. Especially in pediatric audiology, frequency-specific ABR is widely used as young children are incapable of performing valid pure-tone audiometry. In addition to the technique of conventional ABR, auditory steady-state responses (ASSR) are available for frequency-dependent hearing threshold estimation. Although ASSR and ABR are both auditory evoked potentials, both methods have relevant differences: ASSR depend on a peak detection across a spectrum [1], whereas chirp-evoked ABR focus on peak detection of Jewett wave V depending on amplitude and latency [2, 3]. Further, ASSR use a statistical algorithm to detect the hearing threshold whereas the definition of hearing threshold by ABR depends on an examiner reviewing the waveforms and subjectively determining the response.

In the literature, both techniques are widely compared to each other and also to other methods of hearing threshold estimations such as behavioral measures. ABR and ASSR principally show strong correlations in hearing threshold estimations [4–15]. Particularly in profound hearing loss, an increased diagnostic value has been reported for ASSR [16–18]. However, in the case of cochlear nerve hypoplasia with no measurable ABR threshold, a discrepancy between ABR and ASSR hearing threshold estimation has been reported [19]. In a previous study, it could be shown that cochlear nerve hypoplasia itself or in combination with cochlear malformation seems to be associated with significantly greater differences between ABR- and ASSR-derived hearing thresholds estimations [20]. Apart from a rather small sample size, another limitation of the study was that cochlear nerve hypoplasia predominantly presented as single-sided hearing loss whereas most other patients of the study population were affected by bilateral hearing impairment.

Therefore, the present study aimed to compare hearing threshold estimation derived from narrow-band Claus Elberling (CE)-chirps^®^ evoked ABR and ASSR in children all affected by single-sided deafness (SSD) and with varying MRI findings at the impaired ear (cochlear malformation (CM) vs. cochlear nerve malformation (CNM) vs. combined malformation (CM + CNM) vs. absent temporal bone and inner ear pathology).

## Methods

### Patient data

The diagnostic consistency between ABR and ASSR using narrow-band-chirp-stimuli was retrospectively analyzed in children with SSD of varying etiology. SSD was defined as severe-to-profound hearing loss in one ear and normal hearing in the other, as determined by ABR threshold estimations at 1000, 2000, and 4000 Hz. In at least two of these three frequencies, the unilateral hearing loss had to be ≥ 90 dB and ≥ 85 dB in the remaining third frequency. On the contralateral, normally hearing side, ABR threshold estimations at 1000 Hz, 2000 Hz, and 4000 Hz had to be ≤ 25 dB. In addition to same-day measurements of ABR and ASSR, magnetic resonance imaging (MRI) was an obligatory inclusion criterion to identify potential anatomic pathologies. In some children, computer tomography (CT) was also conducted additionally.

Between 01/2014 and 12/2024, 60 children (26 girls and 34 boys) met all above-mentioned inclusion criteria. Of the 60 children, 13 were excluded due to premature birth and/or pre-existing diseases (e.g. trisomy 21 or developmental delay). The remaining 47 children (22 girls and 25 boys) aged from 7 months to 6 years had no further health issues apart from abnormal audiometric findings. In 17 cases, the right ear was affected by SSD, and in the remaining 30 cases the left side.

The retrospective study described was performed in accordance with the ethical principles stated in the Declaration of Helsinki. It was approved by the local ethics committee (project number 25–0149) and the local data protection commissioner. After the collection, all data were anonymized prior to analysis.

### ABR and ASSR registration

In every child, ABR and ASSR data sets were acquired on the same day during general intravenous anesthesia which had been necessary for diagnostic radiology exams by MRI and partially additional CT. In a noise-absorbing and electrically shielded room, ABR and ASSR measurements were obtained consecutively. As stimuli, standard narrow-band CE-chirps^®^ with center frequencies (CF) at 1000 Hz, 2000 Hz, and 4000 Hz were used for both methods. Measurements were performed consecutively with the Interacoustics Eclipse EP25 ABR system (Middelfart, Denmark), as previously described in detail [20]: Calibrated outputs with Etymotic Research Eartone 3 A ABR insert earphones (Elk Grove Village, USA) were used. For recording surface electrodes were positioned on both mastoids, on the high forehead, and the low forehead. The impedance between the electrodes did not exceed 2 kΩ. To prevent interferences from the mains power supply, the hardware high-pass filter of the eclipse was set to 100 Hz with a slope of 12 dB per octave. Further, the signal was low-pass filtered with a cut-off frequency of 3000 Hz to reduce high-frequency interferences, which are outside the range of interest. Further, the absolute value of the electroencephalography (EEG) signal amplitude had to be below 40 µV. The acceptance criteria of a specific frequency and level combination were either 4000 collected EEG recordings or a residual noise level beneath 30 nV, as earlier described [20]. The registration protocol for ABR measurement was also adopted from this previous study, with one modification: Taking the inclusion criterion of SSD into account, the contralateral masking level was set 20 dB below the stimulation level to reduce artifacts from the contralateral ear as much as possible. Again, all ABR measurements were analyzed independently by two experienced audiologists. Using the ABR technique, the hearing threshold was determined based on the detection of the reproducible Jewett wave V.

ASSR were measured as soon as ABR were obtained. In 19 children, ASSR measurements were performed in both ears, and all CFs simultaneously starting at 80 dB nHL according to ABR measurements. In the remaining 28 children, ASSR responses were only collected in the impaired ear with a standard masking level of 70 dB on the contralateral ear.

ASSR were acquired by using the multiple auditory steady-state response (MASTER) technique of the Interacoustics Eclipse Software without automatic threshold correction. If the absolute value of the residual noise exceeded 40 µV, the recordings were rejected. As described earlier in detail [20], the stimuli were modulated and presented at a repetition rate of around 90 Hz (setting of the system). If the algorithm detected a positive response reaching an amplitude level within a 95% confidence interval earlier than in 6 min, the stimulus intensity was decreased by 10 dB. If the confidence interval of response was below 50% within 3 min, the test was repeated with a 5 dB increased stimulus intensity. In data sets without a measurable response up to a stimulus level of 100 dB nHL, the hearing threshold was set to 105 dB nHL and therefore, 105 dB nHL was used for calculations.

### Distortion products of otoacoustic emissions

Distortion product otoacoustic emissions (DPOAE) were measured (MADSEN Capella, GN otometrics GmbH, Münster, Germany) and considered positive if present in at last three frequencies.

### Magnetic resonance imaging (MRI) and computer tomography (CT)

Magnetic resonance imaging (MRI) was performed on a 3 T scanner (Siemens MAGNETOM Skyra, Erlangen, Germany) or on a 1.5 T scanner (Siemens Magnetom Aera, Erlangen, Germany or Siemens Symphony, Erlangen, Germany). In all examinations, the voxel size was 0.5 × 0.5 × 0.5 mm. Further parameters were kept as previously described in detail [20].

As also described earlier in detail [20], computer tomography (CT) was performed on a Dual Source CT-scanner (Siemens SOMATOM Definition Flash), a 2 × 128 row CT scanner with a rotation time of 0.28 s, using a dedicated temporal bone scan protocol with a slice thickness of 0.75 mm, 120 kV, variable mAs (111–151 mAs), including selective reconstruction of each temporal bone using a UHR-kernel (V80u3) in three planes. Two independent radiologists classified inner ear malformations according to Sennaroglu et al. [21, 22].

### Data analysis

Data analysis was performed using Excel (Microsoft, Redmond, WA, US), SigmaPlot and SigmaStat (Jandel Corp., San Rafael, CA, US). For descriptive statistics, we used mean values with standard deviation (SD) and median with range. To compare the distribution of categorical data among different subgroups, a chi-square test was performed. Almost all data failed normality testing (Shapiro–Wilk); therefore, the non-parametric Kruskal–Wallis-One Way Analysis has been used for comparison of Δ ABR–ASSR (in dB) among the subgroups and the Mann-Whitney-U Test for pairwise comparison. The Spearman Rank Order Correlation was carried out to calculate correlations between ABR and ASSR hearing thresholds. A *p*-value ≤ 0.05 was judged significant.

## Results

In 47 ears with SSD, ABR- and ASSR value pairs as well as high-resolution MRI of the temporal bone and the brain were available. In 19 of the 47 children, bilateral hearing threshold estimations were not only obtained by ABR, but also ASSR-measurements were performed in the contralateral ear. These 19 ears served as normal-hearing control ears.

The deaf ears were further grouped by the MRI result: no malformation (*n* = 8; 2 female and 6 male, mean age at the day of hearing threshold estimation 3.8 ± 2.0 years, median age 4.2 years, range 12 months to 6.2 years, 3 right/5 left ears), cochlear nerve malformation (hypoplasia or aplasia; CNM, *n* = 24, 12 female and 12 male, mean age 3.2 ± 2.0 years, median age 3.4 years, range 8 months to 6.3 years, 8 right/16 left ears), cochlear nerve malformation combined with cochlear malformation (CNM + CM, *n* = 8, 4 female and 4 male, mean age 2.2 ± 2.1 years, median age 1.2 years, range 7 months to 5.10 years, 4 right/4 left ears), and isolated cochlear malformation (CM, *n* = 7, 4 female and 3 male, mean age 3.7 ± 1.9 years, median age 4.2 years, range 12 months to 5.8 years, 2 right/5 left ears). There were no statistically significant differences in the distribution of age, gender, or side of the affected ear between the subgroups.

**Fig. 1:**
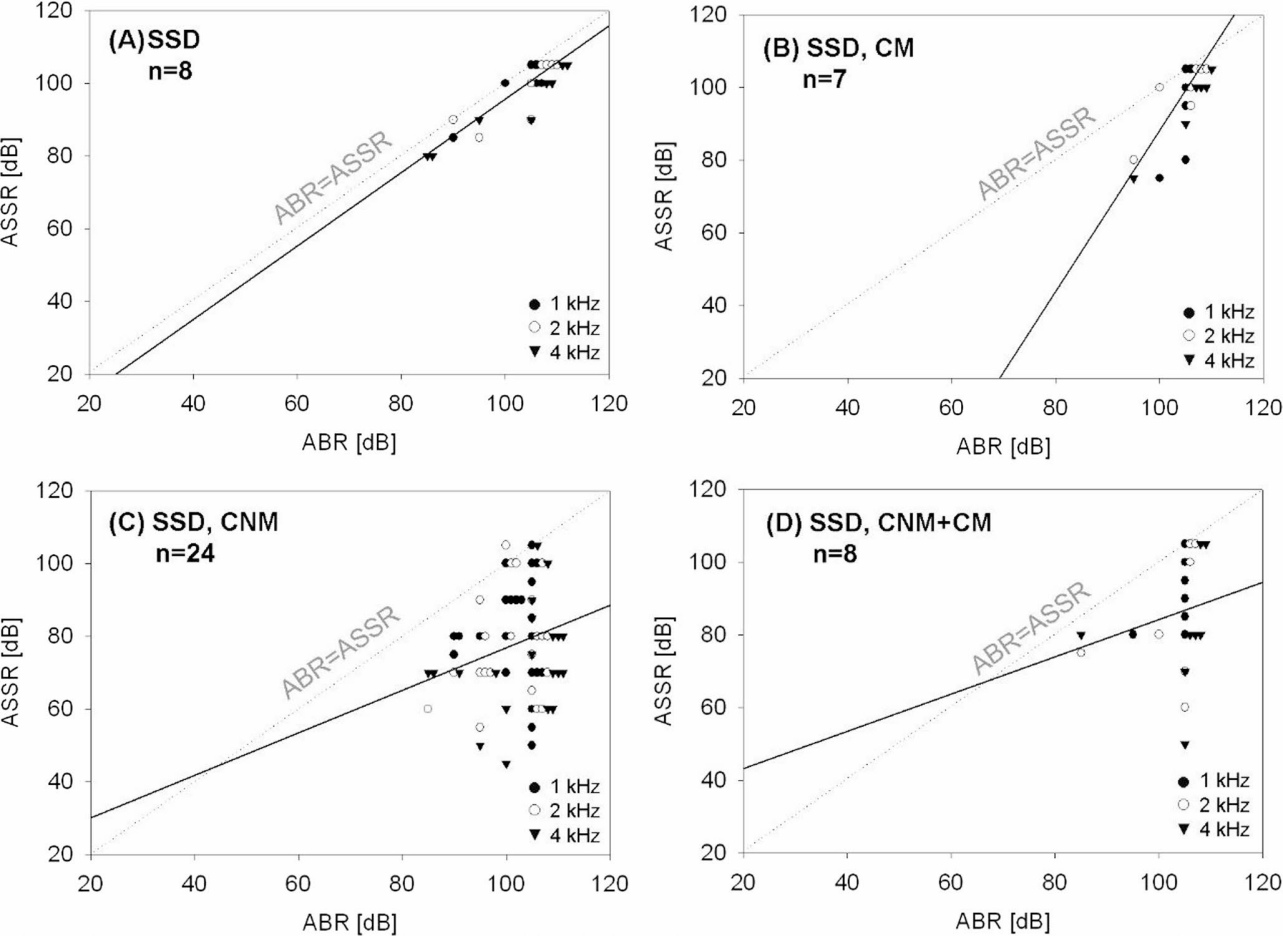
Correlation of ABR (x-axis) and ASSR (y-axis) hearing threshold estimations in dB HL in single-sided deaf ears (**A**) without malformation (*n* = 8; *r* = 0.728, *p* < 0.0001), and (**B**) isolated cochlear malformation (*n* = 7, *r* = 0.574, *p* = 0.011), (**C**) with isolated cochlear nerve anomaly (*n* = 24, *r* = 0.189, *p* = 0.11), and (**D**) with combined cochlear nerve anomaly and cochlear malformation (*n* = 8, *r* = 0.235, *p* = 0.27). Multiple equal value pairs are positioned for their visualization slightly shifted along the x-axis. The continuous line represents the regression line, the dotted line is for orientation (ASSR = ABR hearing threshold estimation)

Overall, a correlation between ABR and ASSR measurements was observed after summarizing value pairs of all CFs in the total study population of 47 ears with SSD (*r* = 0.338, *p* = 0.00005). However, in ears with temporal bone malformation (*n* = 39), the correlation coefficient was much weaker than in the eight deaf ears with normal temporal bone anatomy (*r* = 0.302, *p* = 0.001 vs. *r* = 0.728, *p* = 0.00005). A closer analysis of the different types of temporal bone malformation revealed that a significant correlation between ABR and ASSR measurements could only be found in ears affected by isolated cochlear malformation (*r* = 0.574, *p* = 0.0101). In ears with an anatomically abnormal cochlear nerve, ABR and ASSR measurements correlated only weakly with each other, regardless of the anatomy of the cochlea itself (CNM: *r* = 0.189, *p* = 0.112; CNM + CM: *r* = 0.235, *p* = 0.268). Figure 1 visualizes the different degrees of correlation of ABR and ASSR value pairs between the four cohorts of SSD.

In all 47 deaf ears, ASSR measurements tended to estimate lower hearing thresholds than ABR measurements, but never higher hearing thresholds. In contrast, in the control group of 19 normally functioning ears on the contralateral side, ASSR measurements often revealed slightly higher hearing thresholds than ABR measurements. However, only in one ear, this Δ exceeded − 5 dB. Although in deaf ears, ASSR measurements tended to indicate a lower hearing threshold than ABR measurements, this difference between ASSR and ABR results was heterogeneous between the different sub-groups.

**Fig. 2:**
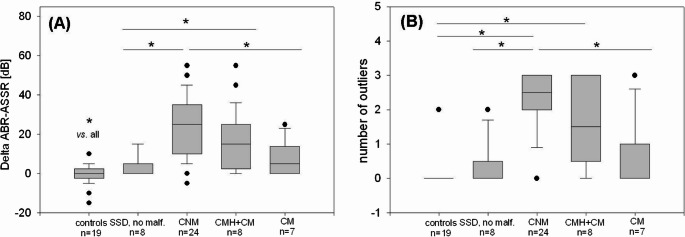
Box plots for each evaluated group (normally hearing ears (controls), deaf ears without malformation (SSD, no malformation), cochlear nerve malformation (CNM), cochlear nerve malformation combined with cochlear malformation (CNM + CM), and cochlear malformation (CM)). In (**A**), the delta between ABR–ASSR hearing threshold estimation (in dB) is plotted with 25% and 75% percentile as the lower and upper edge of the columns, and in (**B**) the number of outliers. *p ≤ 0.05

In order to show the concrete difference between ABR and ASSR hearing threshold estimation, the mean of the difference between ABR and ASSR in dB for 1000 Hz, 2000 Hz and 4000 Hz (Δ ABR-ASSR [dB]) was calculated for each ear. Furthermore, the number of CFs with Δ ≥ 15 dB between ABR and ASSR hearing threshold estimation was counted and defined as outliers. As Fig. 2 shows, both parameters behave quite similarly.

In the 19 healthy control ears, the mean Δ ABR-ASSR [dB] was − 0.36 ± 4.75 dB (*p* = 0.748 ABR vs. ASSR) with an average of 0.11 outliers (median 0, range 0–2). Concerning the 47 deaf ears, the mean Δ ABR-ASSR [dB] was the lowest among the eight ears without any malformation in the MRI (4.38 ± 4.96 dB, *p* = 0.036 ABR vs. ASSR) with an average of 0.38 outliers (median 0, range 0–2). In the seven ears affected by isolated cochlear malformation, the discrepancy was slightly larger (7.63 ± 8.56 dB, *p* = 0.00782) with in average 0.71 outliers (median 0, range 0–3). Solely in ears affected by an anatomically abnormal cochlear nerve, a large difference between ASSR and ABR hearing threshold estimation was found (23.40 ± 15.19 dB, *p* < 0.00001 in CNM vs. 17.08 ± 15.81, *p* = 0.0008 dB in CNM + CM). Also, regarding the outliers, the ears with cochlear nerve anomaly showed clearly different results: on average 2.21 outliers (median 2.5, range 0–3) in CNM and 1.63 outliers (median 1.5, range 0–3) in CNM + CM, respectively.

Table 1 shows the statistical evaluation of intergroup differences performed by Kruskal-Wallis-One way analysis with respect to the concrete Δ ABR-ASSR in dB as well as to the number of outliers (Δ ABR-ASSR ≥15 dB).

**Table 1:** Statistical comparison of the five groups for (A) Δ ABR-ASSR (in dB) and (B) number of outliers (Δ ABR-ASSR ≥15 dB)

(A)	Δ ABR-ASSR [dB]					
Group	Mean	Median	p-value			
			vs. CNM + CM	vs. CM	vs. without malform.	vs. controls
CNM (*n* = 24)	23.40	25	0.072	**< 0.001**	**< 0.001**	**< 0.001**
CNM + CM (*n* = 8)	17.08	15		0.058	**0.047**	**< 0.001**
CM (*n* = 7)	7.63	5			0.327	**< 0.001**
SSD without mal-formation (*n* = 8)	4.38	5				**< 0.001**
Controls (*n* = 19)	−0.35	0				
(B)	Number of outliers (Δ ABR-ASSR ≥15 dB)					
Group	Mean	Median	p-value			
			vs. CNM + CM	vs. CM	vs. without malform.	vs. controls
CNM (*n* = 24)	2.21	2.5	0.290	**0.023**	**< 0.001**	**< 0.001**
CNM + CM (*n* = 8)	1.63	1.5		0.183	0.052	**0.005**
CM (*n* = 6)	0.71	0.5			0.560	0.160
SSD without mal-formation (*n* = 8)	0.38	0				0.440
Controls (*n* = 19)	0.11	0				

All four cohorts with SSD showed significant discrepancies between hearing threshold estimations by ABR and ASSR measurement compared to healthy control ears. Intergroup comparison within the SSD cohort revealed cochlear nerve anomaly as a critical criterion for significantly greater differences between ABR and ASSR results compared to SSD cases with normal cochlear nerve. Isolated cochlear malformations, however, do not result in significantly greater discrepancies between ABR and ASSR values compared to SSD cases without malformations. With regard to the outliers, similar results could be derived from the SSD-intergroup comparison: Only cochlear nerve anomalies lead to a significantly increased number of outliers in the values between ABR and ASSR measurement.

Despite statistical significance in the intragroup comparison between ABR and ASSR estimation in all deaf ears (regardless of the temporal bone anatomy), it has to be critically questioned whether all the above-mentioned differences in hearing threshold estimation are of clinical relevance. Whereas ASSR define an objective hearing threshold owing to a mathematical algorithm, the determination of the ABR response might become difficult especially when approaching the true threshold. Correspondingly, the examiners who subjectively reviewed the waveforms, did not always totally agree whether an ABR response was present or not. Therefore, when calculating the difference between ABR and ASSR hearing threshold levels, a tolerance of +/- 5 dB was applied in the subsequent analysis. ABR and ASSR thresholds were defined as discrepant in case of a discrepancy of ≥ 10 dB. Outliers remained defined as cases with Δ ≥ 15 dB between ABR and ASSR hearing threshold estimation. Table 2 shows a comparison of threshold estimations by ABR and ASSR measurements in more detail.

**Table 2:** Frequency-dependent comparison of ASSR and ABR in different subgroups of radiologic results. In the lowest section, control results of normally hearing contralateral ears are given

	All CFs collapsed		CF 1000 Hz		CF 2000 H		CF 4000 Hz	
CNM								
No. of ears			24	(100%)	24	(100%)	24	(100%)
ASSR = ABR	11	(15%)*<0.00001	4	(17%)*<0.00026	5	(21%)*<0.005	2	(8%)*<0.00002
ASSR < ABR	61	(85%)	20	(83%)	19	(79%)	22	(92%)
ASSR > ABR	0	(0%)	0	(0%)	0	(0%)	0	(0%)
Outliers	53	(74%)*<0.00001	12	(50%)	19	(79%)*<0.00074	22	(92%)*<0.0002
CNM + CM								
No. of ears			8	(100%)	8	(100%)	8	(100%)
ASSR = ABR	9	(38%)*<0.0012	3	(38%)	3	(38%)	3	(38%)
ASSR < ABR	15	(63%)	5	(63%)	5	(63%)	5	(63%)
ASSR > ABR	0	(0%)	0	(0%)	0	(0%)	0	(0%)
Outliers	13	(54%)*<0.0023	4	(50%)	4	(50%)	5	(63%)*<0.039
CM								
No. of ears			6	(86%)	7	(100%)	6	(86%)
ASSR = ABR	12	(63%)	3	(50%)	5	(71%)	4	(67%)
ASSR < ABR	7	(37%)	3	(50%)	2	(29%)	2	(33%)
ASSR > ABR	0	(0%)	0	(0%)	0	(0%)	0	(0%)
Outliers	5	(26%)	2	(33%)	1	(14%)	2	(33%)
SSD without malformation								
No. of ears			8	(100%)	8	(100%)	8	(100%)
ASSR = ABR	20	(83%)	7	(88%)	6	(75%)	7	(88%)
ASSR < ABR	4	(17%)	1	(13%)	2	(25%)	1	(13%)
ASSR > ABR	0	(0%)	0	(0%)	0	(0%)	0	(0%)
Outliers	3	(13%)	1	(13%)	1	(13%)	1	(13%)
Controls (no hearing loss)								
No. of ears			18	(95%)	19	(100%)	19	(100%)
ASSR = ABR	52	(93%)	16	(89%)	18	(95%)	18	(95%)
ASSR < ABR	1	(2%)	1	(6%)	0	(0%)	0	(0%)
ASSR >ABR	3	(5%)	1	(6%)	1	(5%)	1	(5%)
Outliers	2	(4%)	1	(6%)	1	(5%)	0	(0%)

Summarizing all CFs, ASSR and ABR measurements in deaf ears showed equal hearing threshold estimations (+/- 5 dB) in 15% of ears with cochlear nerve hypoplasia vs.38% in ears with cochlear nerve hypoplasia combined with cochlear malformation vs. in 63% in ears with isolated cochlear malformation vs. in 83% in ears without any temporal bone malformation. In control ears without any hearing loss and with normal temporal bone anatomy, the hearing threshold estimations in ASSR and ABR measurements were equal in 93%

*p -value <0.05 vs. SSD without malformation

*CF* center frequency, *CNM* cochlear nerve hypoplasia or aplasia, CNM+CM cochlear nerve hypoplasia oraplasia combined with cochlear malformation,* CM* cochlear malformation, *SSD* single-sided deafness

After the introduction of a tolerance of +/- 5 dB, the picture is clearer: In the 19 normally hearing control ears, ASSR and ABR measurements resulted in equal value pairs in 93% in total. In the control group of SSD without temporal bone malformation, both techniques led to identical estimations only in 83%. However, the difference to normal hearing ears was not statistically significant (*p* = 0.19). Only in deaf ears with an abnormal cochlear nerve, a statistically significant number of discrepant ABR and ASSR measurements was found compared to deaf ears without any temporal bone malformation (see Table 2.). Similar results were observed for outliers (Δ ABR-ASSR ≥ 15 dB): Only in deaf ears with cochlear nerve anomalies, a significant higher number of outliers could be found compared to deaf ears without malformation, as shown in Table 2.. If compared to the 19 normally hearing control ears, all three cohorts with malformation had significantly more outliers, whereas there were no significantly more outliers in the deaf ears without any malformation.

To identify an explanation for the significant discrepancy between ABR and ASSR hearing threshold estimation in cochlear nerve anomaly, several potential influencing factors have been analyzed: (i) residual nerve fibers of the cochlear nerve, (ii) the presence of an enlarged ABR wave I in ABR measurements at low stimulation levels (iii) the presence of otoacoustic emissions.

In three cases, an agenesia of the cochlear nerve was radiologically proven, not only due to the absence of a nerve in MRI (which could also be caused by the limited resolution of the MRI), but also due to a lack of a bony canal between the cochlea and the inner auditory canal which makes the existence of residual nerve fibers rather unlikely in these cases. Nevertheless, the affected ears showed a better hearing threshold estimation by ASSR than by ABR.

**Fig. 3:**
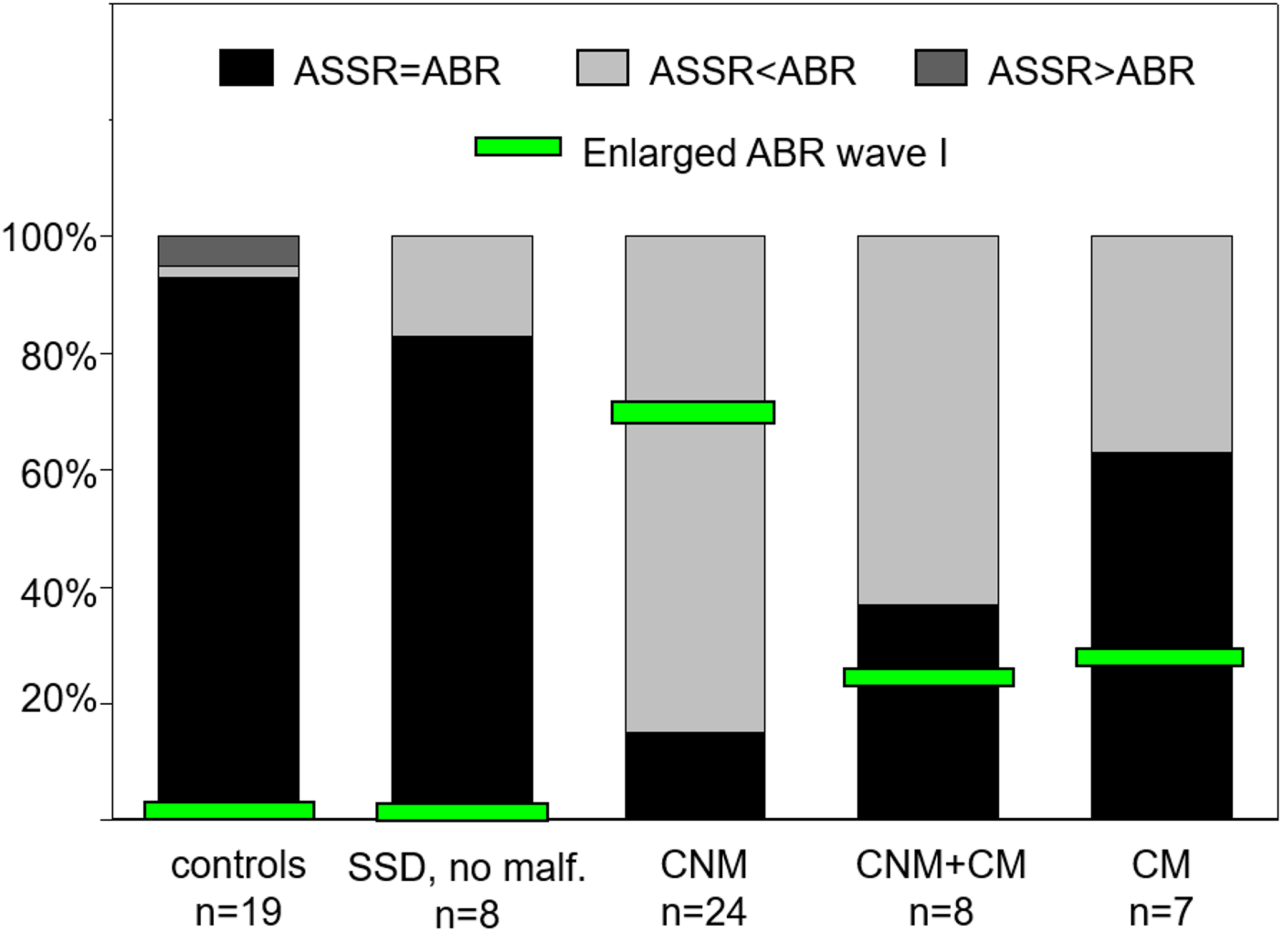
Comparison of ASSR vs. ABR hearing threshold estimation between the five cohorts (see the five greyscale bars) and percentage of enlarged ABR wave I present at lower stimulation levels than wave V (indicated by the green narrow crossbars)

In six children of 24 with CNM (33%) and one child of eight with CNM + CM (12.5%), stable otoacoustic emissions could be detected on the deaf ear (criterion: distortion products of otoacoustic emissions in at last three frequencies). Within the cohort of SSD either without malformation or with isolated cochlear malformation, no patient had otoacoustic emissions detectable. In all seven ears with positive otoacoustic emissions, a high discrepancy between ABR and ASSR levels (Δ ABR-ASSR ≥ 30 dB) was found. In contrast, four further children with a comparably large discrepancy between ABR and ASSR thresholds (Δ ABR-ASSR ≥ 30 dB) had no otoacoustic emissions to be detected. Cochlear microphonic potentials were only recorded in the most recent cases, so a reasonable analysis was not possible. Among the 24 children with CNM, an enlarged ABR wave I was detected in 17 deaf ears (70.8%) at a stimulation level where wave V was no longer detectable. If combined with cochlear malformation, an enlarged ABR wave I was detected in two cases (25%). Of seven deaf ears affected by isolated cochlear malformation, enlarged ABR wave I was also observed in two cases (29%). Only in deaf ears without any malformation and healthy control ears, no enlarged ABR wave I could be detected below the lowest stimulation level for wave V detectability. Figure 3 displays the proportion of enlarged ABR wave I present among the different cohorts and visualizes at the same time the comparison of ABR and ASSR values in all CFs collapsed in each cohort. In all three cohorts with some temporal bone malformation, an enlarged ABR wave I was detectable (indicated by blue bars), whereas no enlarged ABR wave I could be observed in both cohorts without any temporal bone malformation.

## Discussion

The study aimed to investigate the concordance of ABR and ASSR hearing threshold estimation in SSD of different etiology according to MRI. Both in normally hearing control ears (*n* = 19) and in deaf ears without temporal bone malformation (*n* = 8), ABR and ASSR led to equal hearing threshold estimation at a high extent (93 vs. 83%). This good correlation of hearing threshold estimation between the two techniques (ASSR and ABR) has already been described in numerous studies in the literature [9, 14, 18, 23], although some of the studies used varying stimuli and setups in their measurements.

In normally hearing ears, a better concordance of ABR and ASSR values was found with a CF of 2000 Hz and 4000 Hz compared to 1000 Hz. This finding is in line with the literature describing a more accurate prediction of hearing threshold by ASSR with increasing frequency [24–27].

Despite the good concordance between ABR and ASSR values in healthy ears and deaf ears without temporal bone malformation, a significant discrepancy between ABR and ASSR results was found in cases of cochlear nerve anomaly. These data confirm the results of a previous study [20] which was also carried out in our department, however with different patient populations (only three cases of CNM were included in both studies – all remaining 44 cases were uniquely included in the present study). No other studies explicitly investigating hearing threshold estimation by ASSR in patients with CNM were found in the literature search. However, the observation of discrepant values in ASSR and ABR measurement in cochlear nerve deficiency was mentioned in individual cases, although not discussed in detail: Warren and colleagues describe three patients with absent cochlear nerve canal and inconclusive ASSR testing [28]. Ehrmann-Müller and colleagues mention two cases with radiologically confirmed aplasia or hypoplasia of the cochlear nerve in which cochlear implantation was performed based on detectable ASSR potentials and hearing responses in the free field despite questionable ABR responses [19]. In addition, two case reports/series suggest that retrocochlear damage such as severe brainstem dysfunction or multiple sclerosis-related demyelination of the cochlear nerve might not be equally captured by ASSR and ABR and might lead to better results with ASSR than with ABR [29, 30]. The exact cause of the lower (better) ASSR thresholds compared to the ABR measurement was neither analyzed nor discussed in detail.

Initially, when designing the study, we supposed that hearing threshold estimations would differ between techniques because of limited neuronal synchrony. In the ABR technology, multiple very short stimuli are presented, whereby the auditory nerve fibers that encode a certain frequency range should be stimulated as simultaneously as possible. In the ASSR method, continuous stimuli are applied simultaneously across different frequency ranges. While the ABR focus on the reproducible detection of Jewett wave V depending on time and amplitude [2, 3], the ASSR depends on peak detection across a spectrum [1]. This ASSR’s characteristic enables auditory nerve and brainstem potentials to be recorded, even in cases of auditory nerve synchronization disorder [20]. Consequently, the ASSR measurement should estimate a lower (better) hearing threshold than the ABR. According to the present study, the hypothesis that the ASSR method is more robust to synchronization disorders of the auditory nerve, does not fully explain every discrepancy between ABR and ASSR hearing threshold estimation: In the present study, the discrepancies between ABR and ASSR values were particularly strong in cases of isolated malformation of the cochlear nerve. If the nerve anomaly was combined with a malformation of the cochlea, significant discrepancies between ABR and ASSR were still present, but to a less frequent and weaker extent. In cases of isolated cochlear deformity, discrepancies between ABR and ASSR values were even less frequent and weaker, reaching statistical significance only towards healthy control ears, but not compared to deaf ears without malformation. Consequently, the combination of a dysfunctional auditory nerve with preserved cochlear anatomy appears to be a typical feature of cases with significant discrepancies between ABR and ASSR results. Although normal cochlear anatomy does not necessarily imply a normal cochlear function, it must be asked whether a preserved cochlear function despite a malformation of the cochlear nerve may play a decisive role in the discrepancy between the results of the ABR and the ASSR. This hypothesis is also supported by the strong correlation between the two methods in deaf ears without MRI-detectable malformations, where there is likely to be a cochlear damage as reason for deafness that, however, cannot be visualized by MRI. On the other hand, even a normal MRI morphology in deaf ears does not always exclude the possibility that a non-cochlear disorder is the cause of deafness. This could explain why the agreement between ABR and ASSR was 10% worse than in normally hearing control ears. Isolated cochlear malformation is likely to be associated with cochlear dysfunction, consistent with the better correlation between ABR and ASSR. The more frequent detection of otoacoustic emissions and persistent wave I beyond wave V in the cohorts with increased discrepancy between ABR and ASSR also clearly suggests that the better ASSR values may be due to preserved cochlear function.

Cebulla et al. report on one patient with auditory neuropathy spectrum disorder (ANSD) who showed clear ASSR thresholds from 65 dBnHL upwards despite missing click ABR up to 100 dBnHL and suggested that cochlear microphonics evoked by narrow-band chirp stimuli might affect the objective detection of ASSR in patients with ANSD [31].

While our data strengthen this hypothesis, an alternative hypothesis can be weakened at the same time: In cochlear nerve hypoplasia, as opposed to nerve aplasia, residual nerve fibers may allow detection of peak potentials within statistical limits across a spectrum by ASSR, whereas limited synchrony of neuronal function may prevent detection of the same potentials by ABR, since ABR is focused on reproducible detection of Jewett wave V peaks as a function of time and amplitude. However, our study population also included three cases with radiological evidence of cochlear nerve aplasia, in whom ASSR thresholds were still lower than ABR thresholds, which cannot be explained by residual nerve fibers, but apparently by another generator, presumably of cochlear origin.

Against this background, it must be critically examined whether the lower ASSR thresholds reflect the actual hearing thresholds and can realistically enable speech recognition. Verification of the clinical relevance of the discrepant ASSR thresholds is therefore all the more important. Although children affected by SSD are often already older when SSD is diagnosed, most of them are still too young to allow a reliable and valid determination of hearing thresholds using pure tone audiometry with adequate masking. Therefore, a simultaneous clinical examination to determine whether ABR or ASSR thresholds better reflect hearing threshold is not feasible. With increasing age, such an examination of our study population will become possible, but without excluding the occurrence of an interim deterioration in hearing threshold compared to the time of the ABR/ASSR measurement. In any case, the present study emphasizes once again that ASSR alone should not be used in cases of suspected deafness, but only in combination with standard ABR.

### Limitations of the study

Some limitations of the study have to be considered: Despite the long observation period of 11 years at a designated large pediatric audiology center, the sample size of some pathologies is very small because the different etiologies of SSD, which is not very common anyway, are not equally frequent. Our finding of 24 cases of isolated cochlear nerve deficiency in 47 children with SSD is consistent with the literature reporting that approximately 50% of children with SSD have cochlear nerve malformations [32, 33].

Furthermore, hearing threshold levels could be obtained only up to 100 dB due to technically limited sound pressure levels, and beyond that, values were set at 105 dB. This imputing technique has already been used by other researchers [14, 20], but it results in a blurred delta between measurements if a hearing threshold cannot be obtained. In addition, adequate masking is crucial in diagnosing SSD. In ASSR, the masking was realized by 70 dB SPL white noise, which might lead to potential artifacts in ASSR. By choosing SSD as an obligatory including criterion in this study, in contrast to the preceding study by Eder and colleagues [20], the potential risk of cross-hearing by the non-test ear as well as potential artifacts in ASSR due to 70 dB SPL masking affects all cohorts. Consequently, the high discrepancy between ASSR and ABR was not a random effect, but a systematic finding in certain patients.

In addition, it would have been interesting to include the ABR thresholds evoked by click stimuli in the comparison, given that click stimuli are widely used and may also result in different thresholds. Unfortunately, click stimuli were only used to a limited extent for time-saving reasons during anesthesia — for example, to determine latency — and not for estimating thresholds via air conduction.

Finally, our study fails to provide information on the clinical relevance of better ASSR values compared to ABR in SSD. There are conflicting results in the literature: Jafari et al. found no significant correlation between ASSR and behavioral thresholds in 16 adults with bilateral ANSD [34], whereas another study mentioned that ASSR thresholds appear to reflect pure tone thresholds according to preliminary observations in 32 children with ANSD [35].

However, our data support the hypothesis that a cochlear mechanism is the source of the discrepancy, making a clinical relevance of the lower ASSR threshold rather unlikely. Future studies in the same patient population using subjective audiometry with adequate masking are planned to fill this gap, although an interim deterioration in hearing function since the diagnosis of ASSR/ABR cannot be ruled out in future testing.

## Conclusion

In the presence of cochlear nerve malformation in SSD, the hearing threshold estimation by ASSR and ABR frequently provides discrepant values, especially when OAEs and an enlarged ABR wave I are detectable. In other etiologies of SSD, discrepancies between ASSR and ABR are less common. The cause of the discrepancy is most likely a cochlear mechanism rather than residual cochlear nerve fibers. Therefore, ASSR should always be supplemented with ABR measurements for SSD.
